# Causes of fever in Gabonese children: a cross-sectional hospital-based study

**DOI:** 10.1038/s41598-020-58204-2

**Published:** 2020-02-07

**Authors:** José Francisco Fernandes, Jana Held, Magdalena Dorn, Albert Lalremruata, Frieder Schaumburg, Abraham Alabi, Maradona Daouda Agbanrin, Cosme Kokou, Abel Ben Adande, Meral Esen, Daniel Eibach, Ayola Akim Adegnika, Sélidji Todagbé Agnandji, Bertrand Lell, Isabella Eckerle, Beate Henrichfreise, Benedikt Hogan, Jürgen May, Peter Gottfried Kremsner, Martin Peter Grobusch, Benjamin Mordmüller

**Affiliations:** 1 Centre de Recherches Médicales de Lambaréné (CERMEL), Albert Schweitzer Hospital, Lambaréné, B.P: 242, Lambaréné, Gabon; 2 Institut für Tropenmedizin, Eberhard Karls Universität Tübingen, Wilhelmstraße 27, 72074 Tübingen, Germany; 3 German Center for Infection Research (DZIF), partner site Tübingen, Tübingen, Germany; 4 Center of Tropical Medicine and Travel Medicine, Department of Infectious Diseases, Division of Internal Medicine, Amsterdam University Medical Centers, location AMC, University of Amsterdam, Meibergdreef 9, 1105 AZ Amsterdam, The Netherlands; 5 Institute of Medical Microbiology, University Hospital Münster, 48149 Münster, Germany; 6 Albert Schweitzer Hospital, Lambaréné, BP: 118, Lambaréné, Gabon; 7 Infectious Disease Epidemiology, Bernhard Nocht Institute for Tropical Medicine, Bernhard-Nocht-Straße 74 D-, 20359 Hamburg, Germany; 8 German Center for Infection Research (DZIF), partner site Hamburg, Hamburg, Germany; 9 Department of Medicine I, Division of Infectious Diseases and Tropical Medicine, Medical University of Vienna, Währinger Gürtel 18-20, 1090 Vienna, Austria; 10 Institute of Virology, University of Bonn Medical Centre, 53127 Bonn, Germany; 11 Pharmaceutical Microbiology, University Hospital Bonn, University Bonn, 53115 Bonn, Germany

**Keywords:** Diseases, Medical research

## Abstract

The causes of infections in pediatric populations differ between age groups and settings, particularly in the tropics. Such differences in epidemiology may lead to misdiagnosis and ineffective empirical treatment. Here, we investigated the current spectrum of pathogens causing febrile diseases leading to pediatric hospitalization in Lambaréné, Gabon. From August 2015 to March 2016, we conducted a prospective, cross-sectional, hospital-based study in a provincial hospital. Patients were children ≤ 15 years with fever ≥ 38 °C and required hospitalization. A total of 600 febrile patients were enrolled. Malaria was the main diagnosis found in 52% (311/600) patients. Blood cultures revealed septicemia in 3% (17/593), among them four cases of typhoid fever. The other causes of fever were heterogeneously distributed between both bacteria and viruses. Severe infections identified by Lambaréné Organ Dysfunction Score (LODS) were also most often caused by malaria, but children with danger signs did not have more coinfections than others. In 6% (35/600) of patients, no pathogen was isolated. In Gabon, malaria is still the major cause of fever in children, followed by a bacterial and viral disease. Guidelines for both diagnosis and management should be tailored to the spectrum of pathogens and resources available locally.

## Introduction

Causes of fever in African pediatric populations are more diverse than previously thought. A landmark study conducted in Tanzania showed that due to a change in epidemiology, a broad spectrum of pathogens replaced *P. falciparum* malaria as the most common cause of disease in children in this area^1^. However, a few years later, *P. falciparum* malaria, is still seen to be the main cause of febrile illnesses in Ghana, West Africa^2^. When unaware, these differences in epidemiology might lead to misdiagnosis as well as inefficient treatment by the medical personnel. The process of medical diagnosis includes the joint interpretation of symptoms, clinical signs and laboratory findings. Careful selection and prioritization of a diagnostic setup are informed by *a priori* knowledge of the seasonal, local and worldwide frequency and distribution of a given disease^3,4^.

Our study describes the distribution of infections, co-infections, and co-morbidities in children hospitalized for febrile illnesses at the Albert Schweitzer Hospital (HAS) in Lambaréné, Gabon, as an example for a hospital in a semiurban Central African region. In addition, we present the current spectrum of pathogens causing severe disease identified by Lambaréné Organ Dysfunction Score (LODS) in these children.

## Results

### Study patients

A total of 600 febrile patients ≤ 15 years were enrolled in our study. Of these, 280 (47%) were females; 69% (415/600) patients were < 5 years, and median (IQR) age was 29 [12–68] months (Table 1). Seven percent (40/549, NA = 51) had at least one known chronic medical condition prior to admission, among the main ones: 4% (23/600) patients had homozygous sickle cell disease; 1% (6/600) were HIV positive. Vaccination coverage of the expanded program on immunization (EPI) vaccines was above 80% for scheduled doses of BCG, poliomyelitis and pentavalent (diphtheria, pertussis, tetanus, hepatitis B and *Haemophilus influenzae* type b) vaccines, and 54% and 55% for measles and yellow fever vaccines, respectively (Supplementary Fig. S1).

**Table 1:** Characteristics of 600 febrile children enrolled in the study.

Variables	Value
Temperature (°C) Median (IQR)	39 (38.4–39.7)
Weight (kg) Median (IQR)	12 (8.5–18.2)
Age (Months) Median (IQR)	29 (12–68)
Male	320 (53.3%)
Female	280 (46.7%)
Age <12 Months	148 (24.6%)
Age 12–36 Months	182 (30.3%)
Age 36–60 Months	85 (14.2%)
Age >60 Months	185 (30.9%)
Severe anemia*	41 (6.8%)
Sickle cell anemia	23 (4.2%)
Normal weight for age	468 (78%)
Abnormal^ǂ^ weight for age ( < 2 SD mean)	132 (22%)
Duration of hospitalization (days) Mean (SD)	5 (3)

(*) hemoglobin level < 5 g/dL or hematocrit < 15%.

**(ǂ)** weight for age Z-score < −2 standard deviations mean.

**SD**: *standard deviation*.

**IQR**: *interquartile range*.

#### General condition

On admission, 4% (23/593, NA = 7) patients were prostrated. Malnutrition – defined by a weight-for-age Z-score < −2SD was found in 22% (132/600) patients. Table 2a depicts differences of clinical parameters that werefound statistically significant, in relation to major clinical signs.

**Table 2:** Differences in biomedical parameters among study patients in relation to major clinical signs.

(2a) Differences in clinical parameters among study patients in relation to major clinical signs								
Clinical features		All participants	Status	Age (Months)	Body temperature (°C)	Number of stools	Respiratory rate (per minute)	
		N (%)		Value	Value	Value	Value	
General signs	Restless	182 (30.6)	Absent	—	—	—	**44**	
			Present	—	—	—	**49**	
	Wasted	63 (10.6)	Absent	—	—	**1**	—	
			Present	—	—	**0.7**	—	
HEENT	Rhinorrhea	159 (27)	Absent	—	—	—	**44**	
			Present	—	—	—	**50**	
Gastrointestinal	Vomiting	326 (54.3)	Absent	—	—	**0.6**	—	
			Present	—	—	**1.3**	—	
	Diarrhea	188 (32)	Absent	—	—	**0**	—	
			Present	—	—	**3**	—	
	Bloody stool	69 (12)	Absent	—	—	**0.6**	—	
			Present	—	—	**3**	—	
	Abdominal pain	109 (18.4)	Absent	—	—	—	**47**	
			Present	—	—	—	**41**	
Neurological	Prostrated	23 (4)	Absent	—	39	—	—	
			Present	—	38.6	—	—	
	Unconscious	66 (11)	Absent	47		—	—	
			Present	36		—	—	
Respiratory	Frequent sneezing	6 (1)	Absent	—	39	—	—	
			Present	—	38.3	—	—	
	Cough	270 (46)	Absent	—	—	—	**42**	
			Present	—	—	—	**50**	
	Bronchial breath sounds	105 (18.4)	Absent	—	—	—	**44**	
			Present	—	—	—	**53**	
	Flaring	63 (11)	Absent	—	—	—	**44**	
			Present	—	—	—	**58**	
	Sore throat	5 (0.8)	Absent	—	—	—	**45**	
			Present	—	—	—	**61**	
Urinary	Pain in passing urine	14 (2.4)	Absent	—	—	—	**46**	
			Present	—	—	—	**36**	
	Increased frequency of urination	11 (1.9)	Absent	**47**	—	—	—	
			Present	**20**	—	—	—	
**(2b) Differences in biochemical parameters among study patients in relation to major clinical signs**								
**Clinical features**		**All participants**	**Status**	**Hemoglobin (g/dL)**	**Hematocrit (%)**	**Leukocytes (x10**^**9**^**/L)**	**Thrombocytes (x10**^**9**^**/L)**	Transaminases* (IU/L)
		**N (%)**		**Value**	**Value**	**Value**	**Value**	**Value**
General signs	Restless	182 (30.6)	Absent	9.1	26.2	11.9	**216**	33.7
			Present	9.1	26.7	13.2	**280**	33.4
	Lethargic	499 (83.9)	Absent	**9.9**	**28.9**	12.6	**297**	—
			Present	**8.9**	**25.9**	12.2	**224**	—
	Wasted	63 (10.6)	Absent	**9.2**	**26.6**	12.2	239	33
			Present	**8.3**	**24**	13.0	212	37.7
HEENT	Rhinorrhea	159 (27)	Absent	9.2	26.2	11.8	**221**	34.8
			Present	8.4	27	13	**275**	30.9
Gastro-intestinal	Vomiting	326 (54.3)	Absent	**8.8**	**25.6**	12.9	252	34.4
			Present	**9.3**	**27.1**	11.7	223	32.6
	Diarrhea	188 (32)	Absent	**8.8**	**25.6**	12.3	**210**	33.9
			Present	**9.6**	**27.9**	12.1	**292**	31.3
	Abdominal pain	109 (18.4)	Absent	9.1	26.4	12.3	239	33.4^¥^
			Present	9.1	26.1	11.9	218	32.6^¥^
	Hepatomegaly	199 (35)	Absent	**9.5**	**27.8**	11.6	**268**	**55.3**^**ǂ**^
			Present	**8.1**	**23.5**	13.4	**175**	**67.6**^**ǂ**^
**(2b) Differences in biochemical parameters among study patients in relation to major clinical signs**								
**Clinical features**		**All participants**	**Status**	**Hemoglobin (g/dL)**	**Hematocrit (%)**	**Leukocytes (x10**^**9**^**/L)**	**Thrombocytes (x10**^**9**^**/L)**	Transaminases* (IU/L)
		**N (%)**		**Value**	**Value**	**Value**	**Value**	**Value**
Neurological	Convulsion	80 (13.5)	Absent	9.1	26.6	13.3	**244**	32.1
			Present	8.6	24.6	11.9	**186**	40.6
	Prostrated	23 (4)	Absent	9.1	26.4	12.2	237	33.6
			Present	8.6	24.8	13.5	211	32.6
	Unconscious	66 (11)	Absent	9.1	26.6	12.2	**244**	32.9
			Present	8.5	24.4	12.8	**175**	38.9
Respiratory	Frequent sneezing	6 (1)	Absent	9.1	**26.4**	12.2	**234**	**33.8**
			Present	10.9	**35**	12.4	**447**	**22.3**
	Cough	270 (46)	Absent	9	26.1	**11.4**	**201**	**54.9**
			Present	9.2	26.8	**13.08**	**278**	**65.4**
	Crackles	66 (11.5)	Absent	9	26.2	**11.7**	**227**	32.7
			Present	9.6	27.9	**14.7**	**306**	37.7
	Bronchial breath sounds	105 (18.4)	Absent	9	26.4	**11.4**	**221**	33.8
			Present	7.6	26.7	**14.3**	**300**	16
	Flaring	63 (11)	Absent	9.1	**26.7**	**11.7**	234	32
			Present	8.8	**24.4**	**15.2**	246	51.2
	Sore throat	5 (0.8)	Absent	**9**	**26.4**	12.1	234	33.8
			Present	**10.7**	**34.2**	15.4	**447**	23
Urinary	Pain in passing urine	14 (2.4)	Absent	9.1	26.4	**12.2**	**239**	—
			Present	9.4	25.7	**8.3**	**137**	—
	Increased frequency of urination	11 (1.9)	Absent	9.1	26.5	12.1	**241**	**32.9**
			Present	8.7	24.5	10.3	**112**	**21**
Lymphatic	Splenomegaly	253 (44.2)	Absent	**9.9**	**28.8**	12.1	**296**	**53.6**^**ǂ**^
			Present	**7.9**	**23.1**	12.4	**161**	**66.9**^**ǂ**^

HEENT: head, eyes, ears, nose, and throat;

**Values in bold**: statistically significant (the actual p-values are provided in the Supplementary Table S8)

(*) Transaminases: are reflecting ALT values except when specified by a “ǂ” it is rather AST: Aspartate aminotransferase.

**HEENT:** head, eyes, ears, nose, and throat.

**Values in bold**: statistically significant (the actual p-values are provided in the Supplementary Table S9).

Anemia was more pronounced in severely malnourished patients – having a weight-for-age Z-Score < −3SD, with a hemoglobin concentration of 8.3 g/dL *versus* 9.2 g/dL (p = 0.02) (Table 2b).

### Laboratory values and imaging

Laboratory values were mostly as expected for the respective clinical condition. Table 2b depicts differences of hematological parameters and liver function tests in relation to major clinical signs.

**Figure 1:**
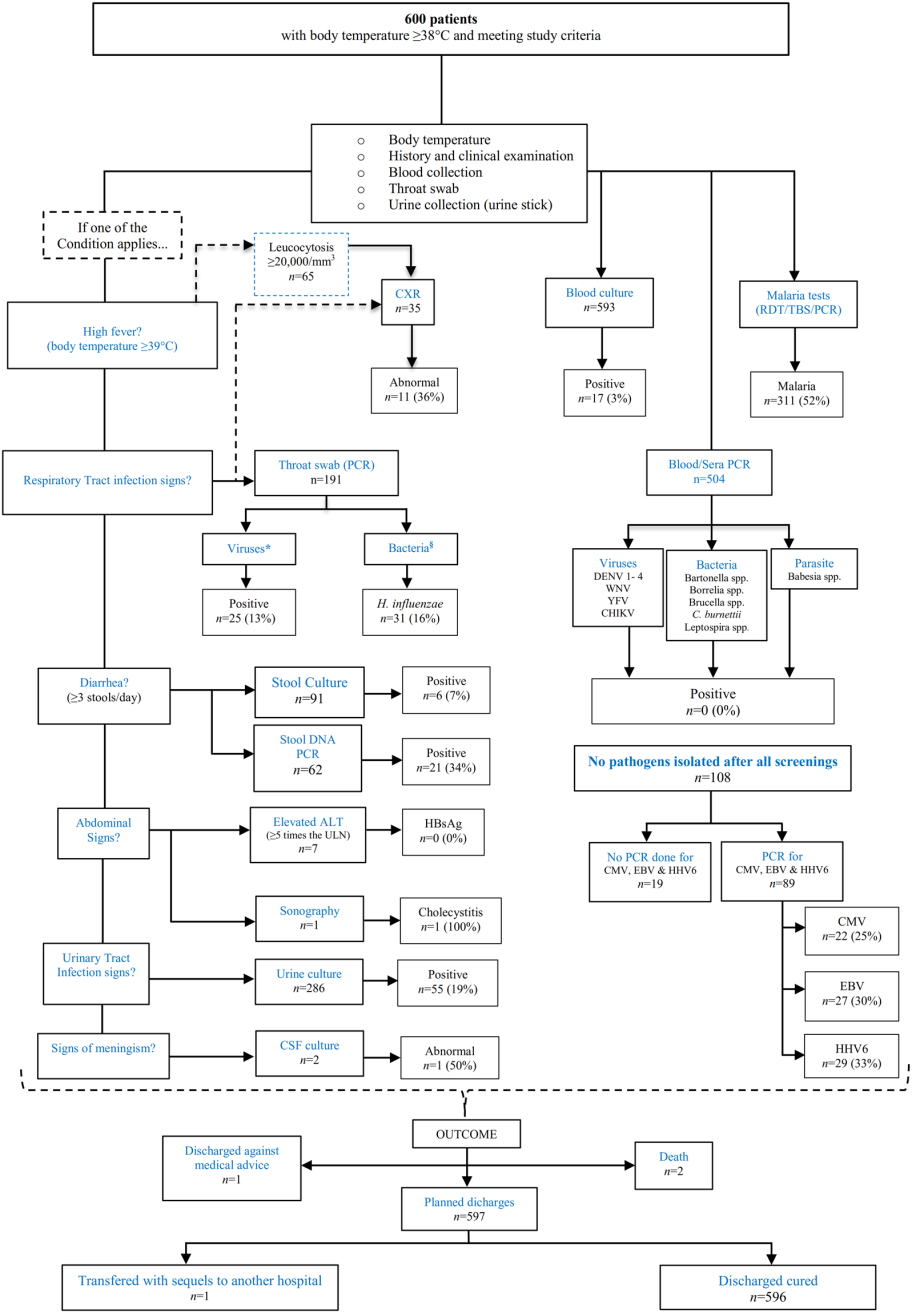
Flow diagram of signs and symptoms, laboratory findings, and diagnoses in all patients throughout the study *: Influenza A & B, Rhinovirus, Enterovirus, Parainfluenza 1–4, Coronavirus (NL63, 229E, OC43 & HKU1), Human metapneumovirus, Respiratory syncytial virus A & B and Adenovirus §: Chlamydia pneumoniae, Haemophilus influenzae, Mycoplasma pneumonia.

Chest radiography was done in children with respiratory signs and/or leucocytes ≥20,000/mm^3^ as early as possible after admission (Fig. 1).

#### Parasitology

Overall, 52% (311/600) patients were diagnosed with *Plasmodium falciparum* (*P.f*.) malaria, ten of them as mixed infections: seven *P. malariae* (*P.m*), three *P. ovale curtisi* (*P.o.c*.), and one *P. ovale wallikeri* (*P.o.w*.). One patient was infected with *P.f*., *P.m*., and *P.o.c*. at the same time.

PCR-screening for *Babesia* spp. was negative in all patients.

Urine analysis revealed one *Schistosoma haematobium* infection.

#### Bacteriology

Blood cultures were taken from 593/600 patients, with 3% (17/593) positives; 4/17 positives were diagnosed with typhoid fever. Pathogens identified in blood cultures are given in Table 3(a). Analyses of pus from two patients requiring abscess drainage revealed *S. aureus* in one and *K. pneumoniae* in the other. Bacteria from urine and stool samples are depicted in Table 3(b,c), respectively. Cerebro-spinal fluid (CSF) cultures were performed in two participants; both were negative after 48 hours of culture. 504 (NA = 96) EDTA blood samples subjected to PCR-screening for *Brucella* spp., *Leptospira* spp., *Bartonella* spp., *Borrelia* spp., *Coxiella burnetii* and *Rickettsia* spp. were found negative. We also screened for *Chlamydiae*, *Haemophilus influenzae*, and *Mycoplasma pneumoniae* in pharyngeal swabs by PCR. Only *H. influenzae* was found in 16% (31/191) patients (Fig. 1).

**Table 3:** Distribution of bacteria by class and site of sampling.

BACTERIA					
(a)	BLOOD	(b)	URINE	(c)	STOOL
PATHOGENS	N (%)	PATHOGENS	N (%)	PATHOGENS	N (%)
**Gram-negative**		**Gram-negative**		**Gram-negative**	
*Enteropathogenic E. coli (EPEC)*	1 (0.2)	*Acinetobacter baumannii*	1 (0.3)	*Group D Salmonella*	1 (1.1)
*Escherichia coli Type III*	1 (0.2)	*Enterobacter cloacae*	2 (0.7)	*Klebsiella pneumoniae*	1 (1.1)
*Group D Salmonella*	1 (0.2)	*Escherichia coli*	26 (9.1)	*Salmonella enterica durham*	1 (1.1)
*K. pneumoniae ESBL*	1 (0.2)	*Escherichia coli ESBL*	2 (0.7)	*Shigella ssp*.	3 (3.3)
*S. enterica enteritidis*	2 (0.3)	*Klebsiella oxytoca*	1 (0.3)	**Negative**	85 (93.4)
*S. enterica typhimurium*	2 (0.3)	*Klebsiella pneumoniae*	7 (2.4)	**Overall**	91 (100)
*Escherichia coli Type III*	1 (0.2)	*Klebsiella pneumoniae ESBL*	4 (1.4)		
*Shigella ssp*.	1 (0.2)	*Kluyvera spp*.	1 (0.3)		
**Gram-positive**		*Proteus mirabilis*	2 (0.7)		
*Group C Streptococcus*	2 (0.3)	*Proteus penneri*	1 (0.3)		
*Micrococcus luteus*	1 (0.2)	*Proteus vulgaris*	1 (0.3)		
*Staphylococcus aureus*	3 (0.5)	*Ralstonia pickettii*	1 (0.3)		
*Staphylococcus saprophyticus*	1 (0.2)	*Salmonella enterica enteritidis*	1 (0.3)		
*Streptococcus pneumoniae*	1 (0.2)	**Gram-positive**			
**Negative**	576 (97.1)	*Enterococcus faecalis*	1 (0.3)		
**Overall**	593 (100)	*Group D Streptococcus*	1 (0.3)		
		*Staphylococcus aureus*	2 (0.7)		
		**Contamination**	107 (37.4)		
		**Negative**	124 (43.4)		
		Others*	1 (0.3)		
		**Overall**	286 (100)		

(a): bacteria in blood culture. (b): list of bacteria found in urine. (c): bacteria in stool culture. *: one parasite (Schistosoma haematobium) found in urine.

#### Virology

Hepatitis B serology (HBsAg determination) was performed in seven patients presenting with an at least five-fold increase in alanine aminotransferase (ALT ≥ 225 UI/L); all were HBsAg negative.

A subset of 89/108 samples that tested negative for all pathogens represented in the initial standard panel (Supplementary Table S1) were screened, by PCR, for cytomegalovirus (CMV), Epstein Barr virus (EBV), human herpesvirus 6 (HHV6). Nineteen samples were not tested for technical reasons. Those three targeted herpesviruses were positive in 25% (22/89), 30% (27/89) and 33% (29/89) patients, respectively (Fig. 1). Co-infections of EBV and HHV6 were present in 13 patients. One child was co-infected by three viruses (Table 4(a)). Screening for dengue viruses 1-4 (DENV 1-4), West Nile virus (WNV), yellow fever virus (YFV) and chikungunya virus (CHIKV) was negative (Fig. 1).

**Table 4:** Distribution of viruses by class and site of sampling.

VIRUSES					
(a)	BLOOD	(b)	THROAT	(c)	STOOL
PATHOGENS	N (%)	PATHOGENS	N (%)	PATHOGENS	N (%)
Cytomegalovirus (CMV)	22 (24.7)	Adenovirus	7 (3.7)	Adenovirus	8 (12.9)
Epstein-Barr virus (EBV)	27 (30.3)	Coronavirus OC43	3 (1.6)	Astrovirus	3 (4.8)
Human herpesvirus6 (HHV6)	29 (32.6)	Coronavirus 229E	2 (1.0)	Norovirus	2 (3.2)
**Negative**	11 (12.4)	Enterovirus	3 (1.6)	Rotavirus	7 (11.3)
Overall	89 (100)	Influenza A	3 (1.6)	Sapovirus	1 (1.6)
**Mixed infections**	**N**	Parainfluenza type 2	1 (0.5)	**Negative**	41 (66.1)
HHV6 + EBV	13	Parainfluenza type 3	1 (0.5)	**Overall**	62 (100)
HHV6 + CMV	8	Rhinovirus	3 (1.6)	**Mixed infections**	**N**
HHV6 + CMV + EBV	1	Respiratory Syncytial Virus	4 (2.1)	Astrovirus + Rotavirus	1
		**Negative**	166 (86.9)	Adenovirus + Astrovirus + Rotavirus	1
		**Overall**	191 (100)		
		**Mixed infections**	**N**		
		Coronavirus OC43 + Coronavirus 229E	1		
		Parainfluenza type 2 + Rhinovirus	1		

**(a)**: viruses in blood. **(b):** list of viruses found in pharyngeal swabs. **(c):** viruses found in stool.

Based on clinical suspicion, eight patients were tested for human immunodeficiency virus type 1 and 2 (HIV1&2), and two were found positive for HIV1, 4 patients were already known to be HIV positive on admission. A total of 191 nasopharyngeal specimens underwent PCR analysis for viruses (Supplementary Table S1), Table 4(b) presents the distribution of the 25 (13%) pathogens identified.

Stool samples of 62 patients underwent PCR, and 21 (34%) were positive for gastrointestinal viruses (Table 4(c)).

#### Imaging

Thirty-five chest radiographs were performed. Two showed no abnormalities, three could not be interpreted due to technical constraints, and 30 showed pathological findings. Among these, 33% (10/30) showed radiologic features of pneumonia with one case also presenting with pleural effusion, 3% (1/30) revealed miliary tuberculosis. A majority of 63% (19/30), although showing abnormal features, did not meet the criteria of pneumonia^5^.

One single abdominal sonography was performed and revealed acute calculous cholecystitis treated by surgery (Fig. 1).

### More frequent infections and their characteristics

In malarial patients, the body temperature was higher compared to non-parasitemic patients (39.2 °C *versus* 38.8 °C; p < 0.001). Consequently, malaria was positively associated with fever grade 3 (39.4 °C – 40 °C; adjusted odds ratio for both sex and age (AOR) 3.2 [1.9–5.6]) and negatively associated with fever grade 1 (<38.6 °C; AOR 0.59 [0.4–0.8]) (Supplementary Table S2). Malaria was equally distributed in all age groups. The evolution of the main clinical and biological parameters among study patients in relation to both infections malaria and *H. influenza* are described in Supplementary Table S3.

Laboratory results showed that anemia was not associated with *H. influenzae* infection, whilst the alanine aminotransferase was lower than in uninfected patients by factor 2 (18.9 IU/L *versus* 41.8 IU/L; p < 0.001) (Supplementary Table S3).

The main pathogen causing urinary tract infections, irrespective of sex and age, was *E. coli* in 52% (28/54) patients.

**Figure 2:**
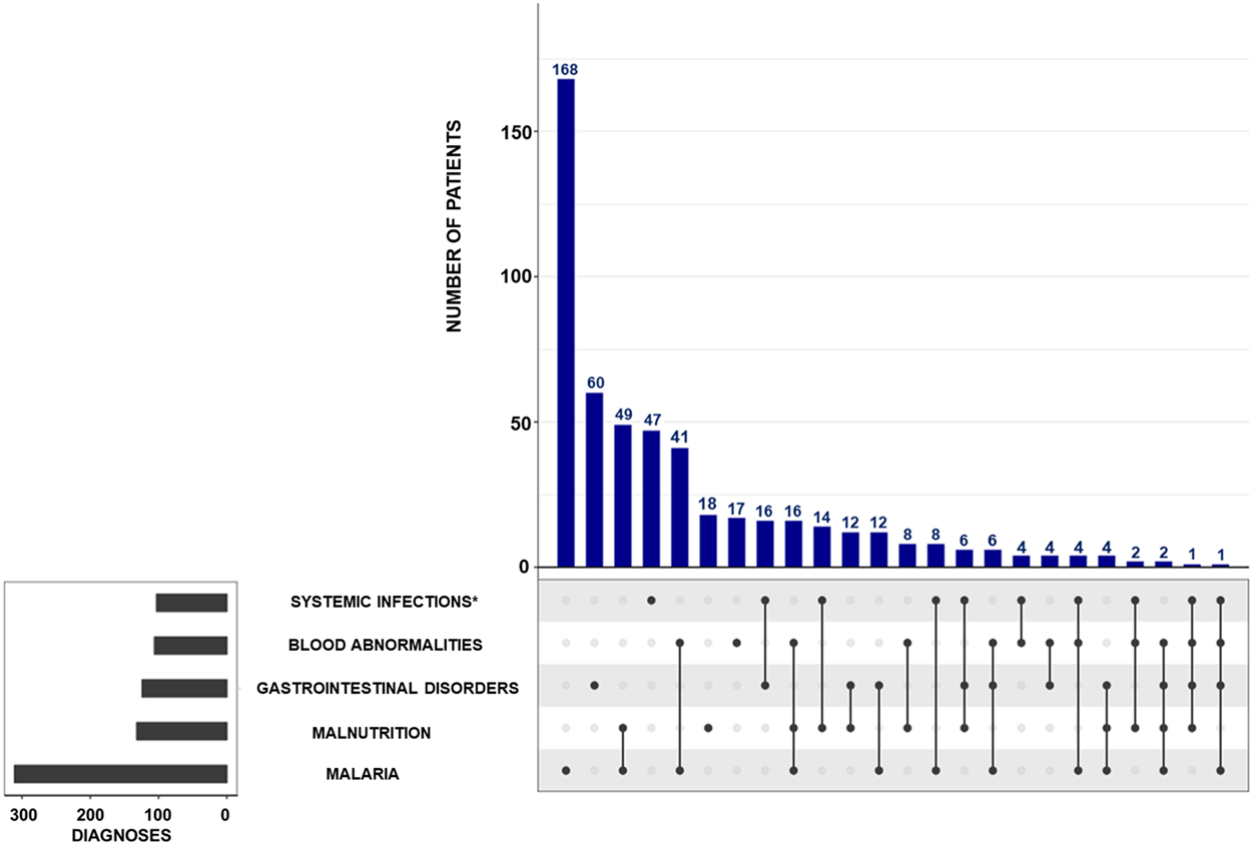
Distribution, frequencies and overlapping of main conditions/diagnoses (according to MedDRA preferred terms) in all 600 patients. ∗Systematic infections other than malaria.

Figure 2 shows the frequency of the main conditions/diagnoses and their co-occurrences; with malaria and malnutrition being the second-most frequent association, seen in 12% (71/600) patients. Systemic infection (bacteria and viruses isolated from blood) occurred together with malaria in 2% (13/600) patients.

All diagnoses and their proportions are listed in Supplementary Fig. S2. In 6% (35/600) of patients, no pathogen was found.

### Children with danger signs

Twelve percent (72/600) children met the criteria of emergency (high risk of death) based on the adapted LODS. Females were 49% (35/72), the median (IQR) age was 19 [10.8–51] months, the nutritional status was normal (weight-for-age Z-score ≥ −2.0) in 79% (57/72) patients. Among those 72 patients, only one child had homozygous sickle cell disease and another one was HIV-positive. On admission, 29% (21/72) patients had a body temperature equal or greater than 39.5 °C, and the most common associated clinical signs were vomiting and convulsions in 53% (38/72) and 42% (30/71, NA = 1), respectively. Severe anemia – hemoglobin <5 g/dL and/or hematocrit <15% – was found in 12% (7/60, NA = 12) patients. Regarding the main diagnoses; severe malaria was found in 47% (34/67, NA = 5) and lower respiratory tract infections (LRTI) in 26% (19/72) patients; the two cases of meningitis and one case of encephalitis were among them. In two patients the cause of fever remained undetermined.

**Figure 3:**
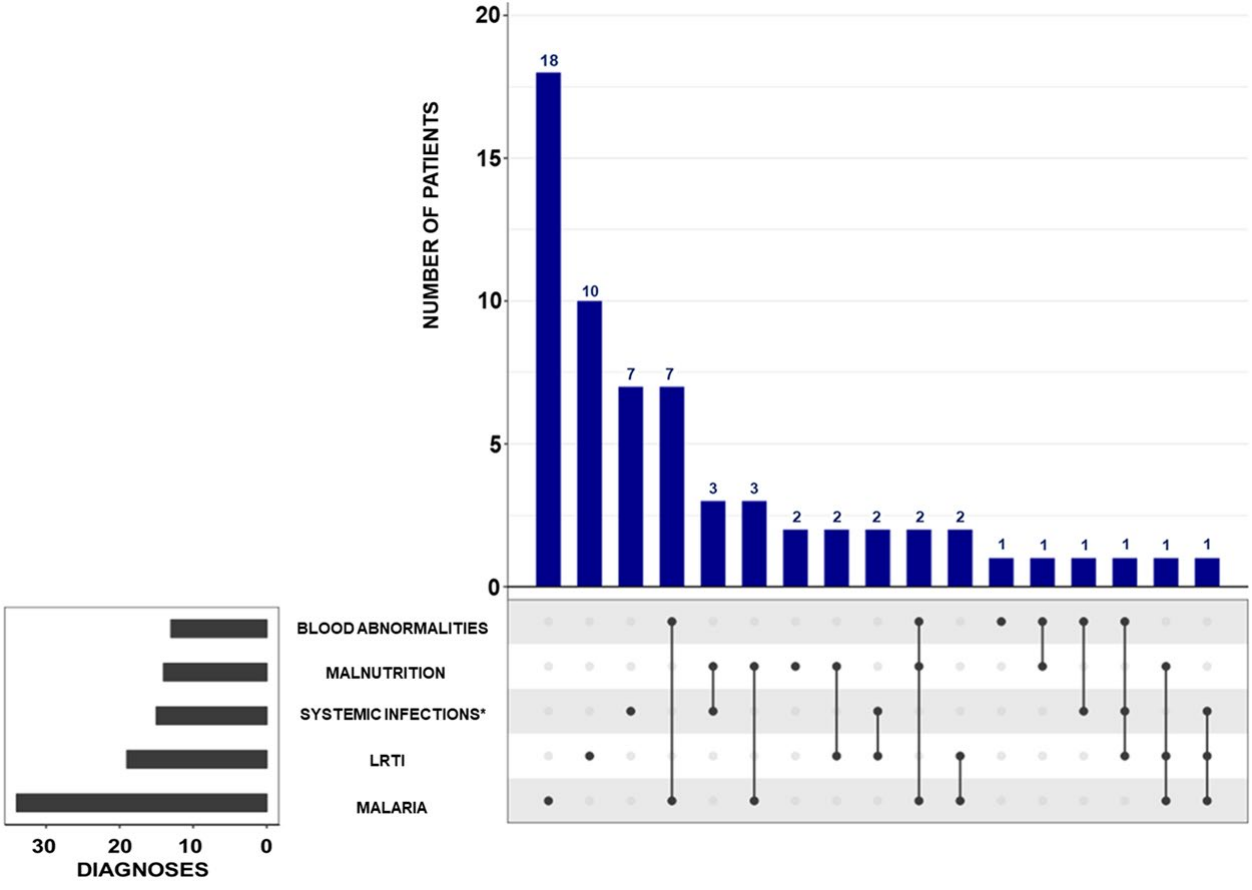
Distribution, frequencies, and overlapping of main conditions/diagnoses (according to MedDRA preferred terms) in patients with danger signs. ∗ Systemic infections other than malaria. LRTI: Lower Respiratory Tract Infections.

Figure 3 shows the frequency of the main diagnoses, including all co-occurrences amongst them, diagnoses coded with MedDRA’s preferred terms are listed in Supplementary Fig. S3.

The two cases of death were part of this group of children presenting with danger signs.

### Outcome

A total of 596 of 600 included patients, were discharged as cured after a mean of five days of hospitalization. Two patients died while in hospital; one of meningitis presenting clinically with meningismus and deep coma, one of pneumonia (clinically suspected pulmonary tuberculosis complicated by bacterial pneumonia). One child, diagnosed with *Staphylococcus aureus* bacteremia, was referred to another hospital after the development of Guillain-Barré syndrome. Another child was taken from the hospital, by his parents, against medical advice (Fig. 1).

Overall, in our study population, neither pre-existing conditions, such as malnutrition or sickle cell anemia nor concomitance of multiple diagnoses were associated with a negative outcome.

## Discussion

In Lambaréné and surrounding villages, malaria remained the leading cause of hospitalization for fever in children, an observation that is in accordance with findings from Ghana^2^ and Burkina Faso^6^. This contrasts with a study from Tanzania where malaria was found only in 10.5% of the patients^1,7^. Consequently, in Lambaréné, infections were most often caused by parasites (62%) (*Plasmodium* species and one *Schistosoma haematobium* infection), followed by bacteria (21%) and viruses (18%). Malaria cases were significantly associated with fever grade 3 (body temperature between 39.4 °C and 40 °C), lethargy, unconsciousness, and convulsions as described in neighboring Congo^8^, and with anemia, malnutrition, and thrombocytopenia. Malnutrition can be an important factor related to malaria as described in hospitalized Mozambican children^9^ and is also pre-disposing to bacteremia^10^. Interestingly, we observed an association of malaria and malnutrition despite the generally low prevalence of malnutrition in Gabon. Anemia is particularly important in malaria-endemic areas because it is the most frequent complication in severe malaria^11,12^.

Respiratory tract infections were the second-most frequent cause of fever in Lambaréné putting young children at risk of respiratory distress, which may be life-threatening^13^, especially where pediatric resuscitation and advanced means of respiratory support are not available. Furthermore, one of the two fatalities that occurred in our study was caused by respiratory failure. Lower respiratory tract infections (LRTI) were the main cause of febrile illness in children, as found in similar studies^14^. These results stress the need for fast and correct malaria diagnosis and radiology in resource-limited areas.

The other identified sources of fever were a heterogeneous group of infections related to pathogens found in blood, urine, stool, and nasopharyngeal specimen.

The relatively large number of CMV by PCR in blood of immune-competent children were most likely not all acute cases even though primary infection with CMV occurs during the first year of life^15^. A certain proportion of the PCR-positives might be explained by delayed sample preparation and consequently, the release of leukocyte-derived CMV DNA^16^. EBV is rather expected to be seen in young adults than in young children^17^. We identified HHV6 in blood by PCR without any exanthema found in these patients possibly due to previous or acute infection without visible skin lesions.

Neither dengue nor chikungunya virus was diagnosed in our study, even though both pathogens were regularly reported in the Lambaréné area^18^. This might be explained by the predominantly sylvatic life cycle of both viruses in the region, where they occur in seasonal small outbreaks^19^.

The proportion of typhoid fever was low (0.7%) compared to a similar study in Tanzania (3.7%)^1^. We acknowledge regional differences in epidemiology and think that continuous improvements in hygiene and sanitation in African communities are further lowering the prevalence of typhoid fever. The same might be the case for bacterial and viral meningitis, which was almost absent (0.2%). This low proportion of meningitis further underlines that not only geographically, Gabon is not part of the West-African meningitis belt^20^. The incorporation of the *H. influenzae* type b vaccine into the Gabonese EPI ten years ago might have contributed to the decline of meningitis cases as seen in Ivory Coast^21^. The 31/191 cases of *H. influenza* found by PCR from pharyngeal swabs were most likely colonization rather than true infections.

Respiratory viruses isolated from nasopharyngeal throat swabs were low in numbers but in accordance qualitatively with what is found in the same age population in other African settings^22^.

*E. coli* was the most frequently isolated pathogen of urinary tract infections, as commonly seen in pediatric populations^23^. In stool samples, viruses were identified less frequently than in Tanzania^1^. In addition, we observed lower rates of invasive bacteria, typhoid and non-typhoid *Salmonella* species in our cohort than in Kenyan children^24^. Those differences may be related to different pathogen dynamics, ecology and level of hygiene between the areas. Co-morbidities were common. In our population, the measles vaccination rate was 54%, well below the WHO^25^ recommended vaccination rate of 95%, in order to prevent measles epidemics and ultimately to eradicate the disease. However, the situation for the other EPI vaccines was better with a vaccination coverage of above 80% for all vaccines that are scheduled to be given within the first 14 weeks of life and not like measles and yellow fever vaccine at 9 months of age.

Therefore, measles need to be considered as one possible differential diagnosis in a proportion of cases even in patients with only unspecific signs and symptoms, *e.g*. lacking characteristic skin eruptions. Pre-existing conditions and co-morbidities, such as sickle cell disease, known HIV-infection and malnutrition had no strong negative impact on the outcome of the febrile diseases, although our methodology might not have been ideal to identify discrete signs.

Malnutrition was present in one-fifth of the patients but did not seem related to immune disorders known to increase the sensitivity of malnourished patients to infections^26^, conversely to what is described from other populations^27,28^.

The cause of fever remained undetermined in 35 patients (6%), similar to the rate of 3.2% reported in Tanzania^1^. Since no control group was recruited the population attributable fractions cannot be estimated. Particularly diseases attributed to herpesviridae and low-grade *P. falciparum* parasitemias may be affected and could increase the proportion of undiagnosed cases, as seen in Ghana^2^.

Based on our results, we recommend that pediatric health care providers should take more care when prescribing antibiotic treatment and if they do, they should base this on the local epidemiology and susceptibility profiles of the bacterial pathogens. Further studies should be designed to assess the relevance of presumptive antibiotic treatment.

The study has some limitations. First, we focused on hospitalized children not considering pathogens from patients that were not admitted for treatment, also a bias towards more severely ill children is present, resulting in different pathogen frequencies, compared to designs targeting outpatients^1^. Secondly, the absence of radiology in our hospital for several months might have distorted the estimation of the prevalence of pneumonia.

This study is one of the few attempts of this kind in Central Africa. Our findings may assist in decision-making which diagnostic tools to apply best in resource-limited settings in relation to the frequency of occurrence of a broader spectrum of pathogens possibly leading to the admission of febrile children in comparable settings.

We described the distribution of pathogens causing fever in children, ill enough to be hospitalized in Lambaréné. We found that malaria remained the most important cause of fever, followed by respiratory tract infections. Both conditions were also the leading cause of severe illness manifestations. We advocate that diagnosis and management should be tailored to the facilities and resources available locally; the use of supportive diagnostic tools (RDTs, microscopy, radiology, and microbiology laboratory) should be strengthened in order to improve clinical care in Lambaréné and similar African settings.

## Methods

### Study design and setting

We report, according to the Strengthening the Reporting of Observational Studies in Epidemiology (STROBE) statement (Table S4)^29^, a systematic, prospective, cross-sectional study in hospitalized children with a fever at the HAS in Lambaréné, Gabon. HAS is one of the two main hospitals of the Moyen-Ogooué province located in the center of Gabon.

### Participants

Children aged ≤15 years, hospitalized at HAS, from August 2015 to March 2016, were included in the study. Apart from consent to participate, the only inclusion criterion was fever (rectal or axillary temperature ≥38 °C). No exclusion criteria were applied.

### Variables

A full diagnostic toolkit to support the identification of infectious agents causing febrile illness was established (Supplementary Table S1).

### Clinical case definitions

Definitions of keywords for characterization of symptoms/signs, medical conditions, and diagnoses were established for harmonization (Supplementary Table S5). To assess severity, we adapted the Lambaréné Organ Dysfunction Score (LODS) focusing on three key items (prostration, coma and chest wall in-drawing)^30^. Prostration was defined by the presence of at least one of the four signs: inability to breast-feed/eat, sit, stand, or walk, depending on child age; whereas coma was defined as Blantyre Coma Score (BCS) ≤ 2.

Symptoms and diagnoses were coded according to the Preferred Terms of the Medical Dictionary for Regulatory Activities (MedDRA) version 19.1 of September 2016 from verbatim descriptions.

### Data sources and bias assessment

Study physicians completed the questionnaire, including sociodemographic and clinical information as presented in Supplementary Table S6.

Based on symptoms and signs, children were assigned to a specific syndrome (Supplementary Table S1) and samples were taken accordingly (Fig. 1). Sample collection was based on two principles: first, to find the immediate diagnosis decisive for treatment based on investigations (*i.e*. rapid diagnostic kits and laboratory-based investigations), routinely available in Lambaréné; second, to allow for diagnosis by advanced techniques carried out in Lambaréné and at the partner institutions presented in Supplementary Table S7.

We tried to minimize the selection biases (*i.e*. admission, volunteer, and non-response bias) common in cross-sectional studies by recruiting almost all febrile hospitalized children in the study period.

### Sample size considerations

This observational study aimed at detecting non-dominant fever-causing pathogens controlling for seasonal variations in disease frequency. The pediatric ward had 1,933 admissions in 2014, of which 69% had fever as the lead symptom. To cover at least one rainy and one dry season and to detect uncommon pathogens, we decided to recruit 600 children over a period of eight months, requiring the inclusion of at least 50% of eligible children; yielding a 95% probability to detect uncommon pathogens (prevalence ≤0.05%).

### Data management and statistical methods

Data were collected by filling a paper questionnaire that was manually entered into an electronic database - Research Electronic Data Capture (REDCap) Software - Version 5.7.2, 2015^31^.

Missing data were handled by list-wise deletion (complete-case analysis). Missing values are reported as “not available”.

Analyses were done with R software V3.5.1 (2018) (www.r-project.org). Continuous exposure data were described and compared according to their distribution. Prevalence ratios and odds ratios were used to show associations among dichotomous variables. Stratification was used to show effect differences amongst a third variable. Corresponding multivariate models were established to account for confounding and interaction.

Results of the bivariable and multivariable analysis were reported as crude and adjusted odds ratio at 95% confidence intervals (95% CI), and statistical significance was defined as a two-sided *p*-value < 0.05.

All methods were performed in accordance with relevant guidelines and regulations.

### Ethics declarations

After being validated by both the Scientific Review Board and the Institutional Ethics Committee of Centre de Recherches Médicales de Lambaréné (CERMEL), our study protocol was submitted to the Gabon National Ethics Committee on 06 February 2015 and obtained an approval (Number 006/2015/SG/P) on 28 February 2015.

Written informed consent was obtained from all parents/legal guardians prior to enrolment. Children aged eight years and above were enrolled only when they agreed and provided their assent form; except for those unconscious, in which case only the informed consent provided by a parent or guardian was considered sufficient. The study team decided upon the medical condition of the patient and determined whether he/she was able to sign the assent.

Two copies of the informed consent form, as well as the assent form, had to be signed. One was kept by the study personnel for documentation, while the second copy was given to the patient’s parents/guardians.

## Supplementary information

Supplementary information.

## Data Availability

All data supporting the findings of this study are available from the corresponding authors upon request.
