# Supplementary material for: Causes of fever in Gabonese children: a cross-sectional hospital-based study
Source: Sci Rep. 2020 Feb 7;10:2080. doi: 10.1038/s41598-020-58204-2 (PMC7005879; doi:10.1038/s41598-020-58204-2)
Supplement: Supplementary file 1 — Supplementary information. [file 41598_2020_58204_MOESM1_ESM.pdf]

Title: Causes of fever in Gabonese children: a cross-sectional hospital-based study

José Francisco Fernandes[1,2,3,4], Jana Held*[1,2,3], Magdalena Dorn[1], Albert Lalremruata[2,3], Frieder Schaumburg[5], Abraham Alabi[1], Maradona Daouda Agbanrin[1], Cosme Kokou[6], Abel Ben Adande[6], Meral Esen[1,2,3], Daniel Eibach[7,8], Ayola Akim Adegnika[1,2,3], Sélidji Todagbé Agnandji[1,2,3], Bertrand Lell[1,9], Isabella Eckerle[10], Beate Henrichfreise[11], Benedikt Hogan[7,8], Jürgen May[7,8], Peter Gottfried Kremsner[1,2,3], Martin Peter Grobusch*[1,2,3,4], Benjamin Mordmüller[1,2,3]

1 Centre de Recherches Médicales de Lambaréné (CERMEL), Albert Schweitzer Hospital, Lambaréné, B.P: 242 Lambaréné - Gabon

2 Institut für Tropenmedizin, Eberhard Karls Universität Tübingen, Wilhelmstraße 27, 72074 Tübingen, Germany

3 German Center for Infection Research (DZIF), partner site Tübingen, Tübingen, Germany

4 Center of Tropical Medicine and Travel Medicine, Department of Infectious Diseases, Division of Internal Medicine, Amsterdam University Medical Centers, location AMC, University of Amsterdam, Meibergdreef 9, 1105 AZ Amsterdam, The Netherlands

5 Institute of Medical Microbiology, University Hospital Münster, 48149 Münster, Germany

6 Albert Schweitzer Hospital, Lambaréné, BP: 118 Lambaréné, Gabon

7 Infectious Disease Epidemiology, Bernhard Nocht Institute for Tropical Medicine, Bernhard-Nocht-Straße 74 D-20359 Hamburg, Germany

8 German Center for Infection Research (DZIF), partner site Hamburg, Hamburg, Germany

9 Department of Medicine I, Division of Infectious Diseases and Tropical Medicine, Medical University of Vienna, Währinger Gürtel 18-20, 1090 Vienna, Austria

10 Institute of Virology, University of Bonn Medical Centre, 53127 Bonn, Germany

11 Pharmaceutical Microbiology, University Hospital Bonn, University Bonn, 53115 Bonn Germany

*Correspondence:

jana.held@uni-tuebingen.de
m.p.grobusch@amsterdamumc.nl

METHODS

Laboratory analyses

Laboratory investigations were conducted on-site at CERMEL's laboratories in Lambaréné (Gabon). Further bacteriological and viral advanced molecular testings were performed in specialized laboratories of partner universities in Hamburg, Bonn, Gießen, Münster, and Tübingen (Germany).

In blood: antigen detecting rapid diagnostic tests were used: Paracheck Pf dipstick (Orchid Biomedical Systems, India) for *Plasmodium falciparum*; Rota/Adeno Kombi Test (Diagnostik Nord GmbH, Germany) for adenovirus and rotavirus; SD Bioline Alere-52FK10 (Republic of Korea) for norovirus; and Vikia HBsAg (Biomerieux SA, France) for hepatitis B virus.

The only antibody detecting rapid test was the Right Sign HIV 1&2 Test (Diagnostik Nord GmbH, Germany) for HIV 1 and HIV2. Whereas, in urine: the OneStep Legionella Urinary Antigen RapidCard (Diagnostic Automation|Cortez Diagnostics INC, Woodland Hills, California, USA) for *Legionella pneumophila*; and urine dipsticks Combur9 Test Cobas (Roche Diagnostic Limited, Switzerland).

All these points of care tests were performed according to manufacturers' instructions.

Thick blood smears were applied for *malaria* microscopic diagnosis. Staining was done by 20 minutes immersion into a 10% Giemsa solution and *Plasmodium* spp. asexual forms were determined by the Lambaréné method as described elsewhere[1]. Urine and stool cultures were performed using the procedures described in the respective

microbiology laboratories specific standard operating procedures (SOPs). A urine culture was considered positive if it yielded ≥100,000 colony forming units per milliliter (CFU/mL). For blood culture, between 1 and 3 mL of blood was collected in Bactec Peds Plus culture bottles and incubated in a Bactec 9050 blood culture system (BD Biosciences). Positive samples were cultured on blood agar, chocolate agar, and cysteine-, lactose-, and electrolyte-deficient agar. Suspicious colonies were subjected to species identification using API-E test strips (bioMérieux, Marcy l'Etoile, France). In Germany, both species confirmation and antimicrobial susceptibility testing were done using Vitek 2 automated test systems (bioMérieux) and EUCAST breakpoints.

Sera, whole blood, nasal and throat swabs were placed in tubes containing RNA*later* stabilization solution (Invitrogen by Thermo Fisher Scientific, Vilnius, Lithuania) for storage and transport.

DNA/RNA Extraction

DNA/RNA extractions were made using commercial kits for serum, CSF and nasopharyngeal swabs (RTP Pathogen Kit, Stratec Molecular GmbH, Berlin, Germany); out of stool (QIAamp DNA Stool Mini Kit Qiagen GmbH, Hilden, Germany) and from whole blood [QIAsymphony DSP DNA Midi Kit automated on QIAsymphony SP robot (SN 34440, Qiagen Hilden, Germany)] according to the manufacturer's instructions, obtaining an elution volume of 100 µL.

PCR for Different Pathogens

Real-time PCR, including subtype differentiation on DNA/ARN from **stool** targeting: *E. histolytica*[2,3], *E. dispar*[2,3], *Cryptosporidium* spp.[2,3], *G. lamblia*[2,3]*;* norovirus[4], astrovirus[4], rotavirus[4], adenovirus[4], sapovirus[4].

Real-time reverse transcription polymerase chain reaction (PCR) assays on DNA/ARN from **nasal/nasopharyngeal swab** for the detection of: Influenza A and B viruses[5], Rhinovirus[5]; human coronaviruses NL63, 229E, OC43 & HKU1[5], Human parainfluenza viruses 1-4[5], human metapneumovirus[5] , respiratory syncytial viruses[5], adenovirus[5], enterovirus[5], *Chlamydiae*[6], *S. pneumoniae*[7], *H. influenzae* (> 10^5 DNA copies/mL)[8]*, M. pneumonia*[9]*.*

Real-time reverse transcription polymerase chain reaction (PCR) assays on DNA/ARN from **the blood** for the detection of *Brucella* spp[10], *Leptospira* spp.[11,12], *Rickettsia* spp.[13], *Bartonella* spp.[14], *Borrelia* spp.[15], *Babesia* spp. (in-house PCR).

Diagnostic methods applied to serum were screening for: alphaviruses[16], flaviviruses[17], human herpesvirus 6 [Human Herpes Virus 6 BHLF3 genesig Standard Kit, Primerdesign Ltd].

Detection of Plasmodium infections and identification of *Plasmodium* species

All samples underwent an initial screening for Plasmodium infection done by Pan-Plasmodium reverse transcription quantitative PCR (RT-qPCR) assay as reported elsewhere[18].

For the identification of malaria species would be processed in two subsequent steps:

Step 1: In order to be used as a template for quantitative real-time PCR (qPCR) assay, all samples initially positive by the Pan-Plasmodium RT-qPCR assay underwent a conventional PCR amplification following a protocol described earlier[19]. The volume for this amplification was set to 50µL containing: 7.5 µL of nucleic acids extract, 300µM of each primer (PLU5 and PLU6), 1X Qiagen PCR buffer with 1·5mM $MgCl_2$, 250 µM dNTPs each, and 1U Taq DNA polymerase (Qiagen, cat #201223). The following cycling

conditions were used: denaturation at 95°C for 5 minutes, then by series of 20 cycles (95°C for 30 s, 58°C for 30 s, and 72°C for 1:20 min) and a final extension at 72°C for 5 minutes.

Step 2: For the diagnosis of *Plasmodium* spp. infections primers and probes *P. malariae, P. ovale curtisi, P. ovale wallikeri,* for *P. falciparum and for P. vivax*[20] were developed for that purpose. The monoplex qPCR assay was performed for the following human malaria species: *P. falciparum, P. malariae, P. ovale curtisi, P. ovale wallikeri, and P. vivax using* the LightCycler 480 Instrument II (Roche Applied Science). Each qPCR assay was made of 2.5µL of the pre-amplified product, 1X SensiMix II Probe No-ROX, 300µM of each primer pair, and 150 µM of each probe per 10µL final reaction volume. The cycling conditions were as follows: polymerase activation for 10 min at 94°C, followed by 45 cycles of (10 seconds at 95°C and for 60 seconds at 60°C). All samples, along with non-template control (NTC) and positive control, were tested in duplicate. The LightCycler 480 software (version 1.5.1.62) automatically calculated and generated the quantification threshold cycle value (C_t) using the second derivative maximum method integrated. Positivity was assessed visually by analyzing the amplification curves for variability between each sample replicates (SD ≤ 1 cycle) and the quantification threshold value less than 40 (C_t <40). All the assays were tested and validated in accordance with MIQE guidelines[21].

Radiology

Chest X-rays were performed on-site in the context of clinical activities performed routinely during pediatric care. All the features considered as abnormal findings were interpreted using the standardized interpretation of pediatric chest radiographs for the

diagnosis of pneumonia in epidemiological studies from the World Health Organization[22]. This x-ray film reading allowed distinguishing diagnoses of radiological pneumonia and non-radiological pneumonia.

REFERENCES

1. Planche, T. *et al.* Comparison of methods for the rapid laboratory assessment of children with malaria. *Am. J. Trop. Med. Hyg.* **65**, 599–602 (2001).
2. Verweij, J. J. *et al.* Simultaneous Detection of Entamoeba histolytica, Giardia lamblia, and Cryptosporidium parvum in Fecal Samples by Using Multiplex Real-Time PCR. *J. Clin. Microbiol.* **42**, 1220–1223 (2004).
3. Verweij, J. J., Laeijendecker, D., Brienen, E. A. T., van Lieshout, L. & Polderman, A. M. Detection of Cyclospora cayetanensis in travellers returning from the tropics and subtropics using microscopy and real-time PCR. *Int. J. Med. Microbiol. IJMM* **293**, 199–202 (2003).
4. Aldabbagh, S., Eckerle, I., Müller, A., Delwart, E. L. & Eis-Hübinger, A. M. Salivirus type 1 and type 2 in patients with acute gastroenteritis, Germany. *J. Clin. Virol. Off. Publ. Pan Am. Soc. Clin. Virol.* **72**, 16–19 (2015).
5. Annan, A. *et al.* Similar virus spectra and seasonality in paediatric patients with acute respiratory disease, Ghana and Germany. *Clin. Microbiol. Infect. Off. Publ. Eur. Soc. Clin. Microbiol. Infect. Dis.* **22**, 340–346 (2016).
6. Lienard, J. *et al.* Development of a new chlamydiales-specific real-time PCR and its application to respiratory clinical samples. *J. Clin. Microbiol.* **49**, 2637–2642 (2011).
7. Carvalho, M. da G. S. *et al.* Evaluation and improvement of real-time PCR assays targeting lytA, ply, and psaA genes for detection of pneumococcal DNA. *J. Clin. Microbiol.* **45**, 2460–2466 (2007).

8. Abdeldaim, G. M. K. *et al.* Detection of Haemophilus influenzae in respiratory secretions from pneumonia patients by quantitative real-time polymerase chain reaction. *Diagn. Microbiol. Infect. Dis.* **64**, 366–373 (2009).
9. Welti, M. *et al.* Development of a multiplex real-time quantitative PCR assay to detect Chlamydia pneumoniae, Legionella pneumophila and Mycoplasma pneumoniae in respiratory tract secretions. *Diagn. Microbiol. Infect. Dis.* **45**, 85–95 (2003).
10. Queipo-Ortuño, M. I. *et al.* Rapid diagnosis of human brucellosis by SYBR Green I-based real-time PCR assay and melting curve analysis in serum samples. *Clin. Microbiol. Infect.* **11**, 713–718 (2005).
11. Mérien, F., Amouriaux, P., Perolat, P., Baranton, G. & Girons, I. S. Polymerase chain reaction for detection of Leptospira spp. in clinical samples. *J. Clin. Microbiol.* **30**, 2219–2224 (1992).
12. Woo, T. H. *et al.* Identification of pathogenic Leptospira genospecies by continuous monitoring of fluorogenic hybridization probes during rapid-cycle PCR. *J. Clin. Microbiol.* **35**, 3140–3146 (1997).
13. Sothmann, P. *et al.* Rickettsia felis Infection in Febrile Children, Ghana. *Am. J. Trop. Med. Hyg.* **96**, 783–785 (2017).
14. Raoult, D. *et al.* First Isolation of Bartonella alsatica from a Valve of a Patient with Endocarditis. *J. Clin. Microbiol.* **44**, 278–279 (2006).
15. Sokhna, C. *et al.* Point-of-Care Laboratory of Pathogen Diagnosis in Rural Senegal. *PLoS Negl. Trop. Dis.* **7**, (2013).

16. Eshoo, M. W. *et al.* Direct broad-range detection of alphaviruses in mosquito extracts. *Virology* **368**, 286–295 (2007).
17. Chao, D.-Y., Davis, B. S. & Chang, G.-J. J. Development of Multiplex Real-Time Reverse Transcriptase PCR Assays for Detecting Eight Medically Important Flaviviruses in Mosquitoes. *J. Clin. Microbiol.* **45**, 584–589 (2007).
18. Mordmüller, B. *et al.* Sterile protection against human malaria by chemoattenuated PfSPZ vaccine. *Nature* **542**, 445–449 (2017).
19. Snounou, G. *et al.* High sensitivity of detection of human malaria parasites by the use of nested polymerase chain reaction. *Mol. Biochem. Parasitol.* **61**, 315–320 (1993).
20. Groger, M. *et al.* Prospective Clinical Trial Assessing Species-Specific Efficacy of Artemether-Lumefantrine for the Treatment of Plasmodium malariae, Plasmodium ovale, and Mixed Plasmodium Malaria in Gabon. *Antimicrob. Agents Chemother.* **62**, e01758-17 (2018).
21. Bustin, S. A. *et al.* The MIQE guidelines: minimum information for publication of quantitative real-time PCR experiments. *Clin. Chem.* **55**, 611–622 (2009).
22. Cherian, T. *et al.* Standardized interpretation of paediatric chest radiographs for the diagnosis of pneumonia in epidemiological studies. *Bull. World Health Organ.* **83**, 353–359 (2005).

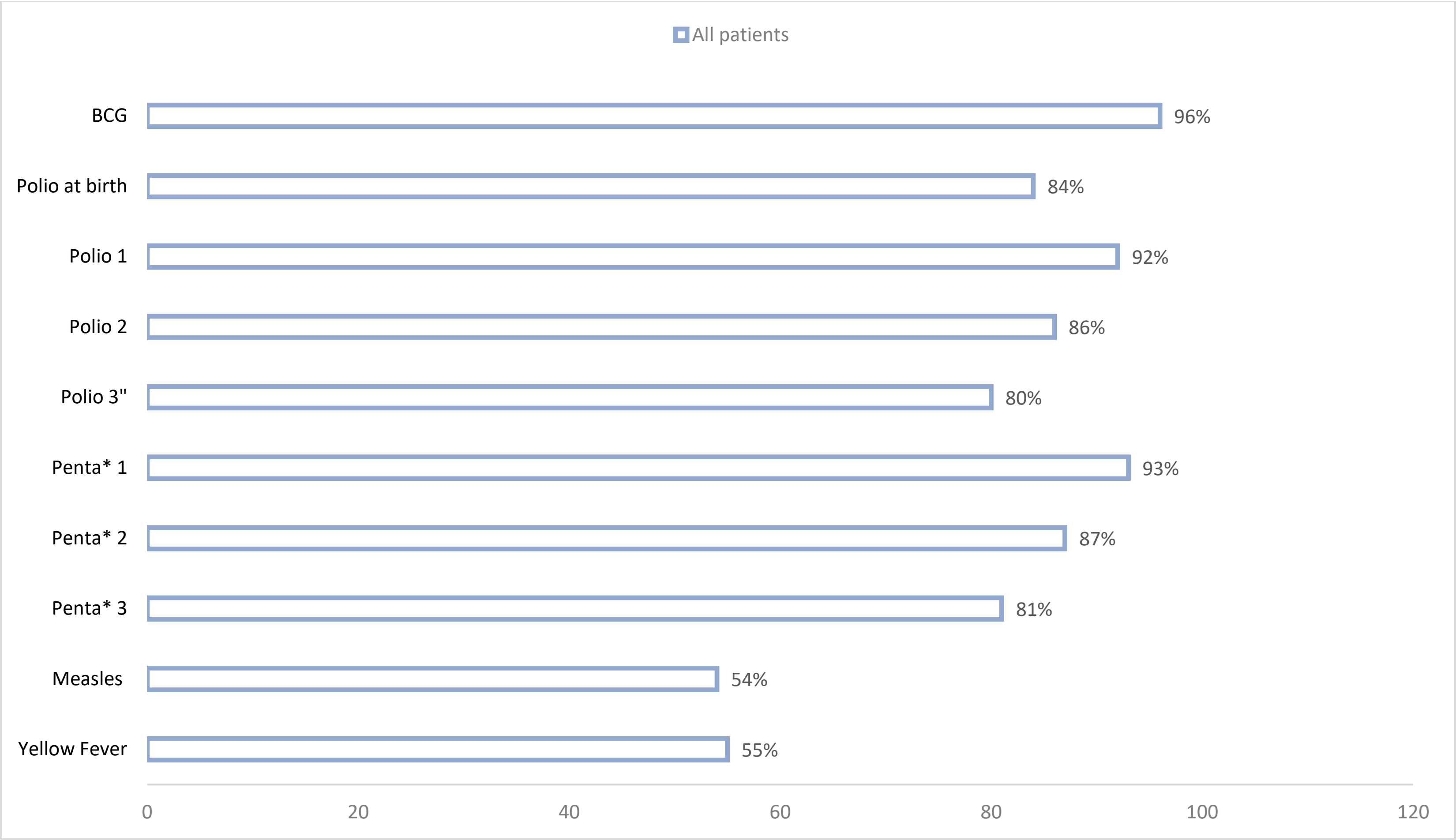

Supplementary Figure S1. Proportions of both all patients and those with danger signs vaccinated with vaccines of the Expanded Programme on Immunization (EPI). **BCG**: Bacillus Calmette–Guérin *vaccine,* used against tuberculosis; **Polio**: Oral Polio vaccine (OPV) used against poliomyelitis, and that consists of a mixture of live attenuated poliovirus strains of each of the three serotypes; **Polio 3"**: combined immunization with both OPV and Injectable Polio Vaccine (IPV: Inactivated virus vaccine); **Penta***: the pentavalent vaccine contains five antigens: Diphtheria Toxoid; Tetanus Toxoid; Whole-cell *Bordetella pertussis*; recombinant Hepatitis B virus surface antigen [HBsAg (rDNA)], and purified capsular *Haemophilus influenzae type b* Polysaccharide (PRP).

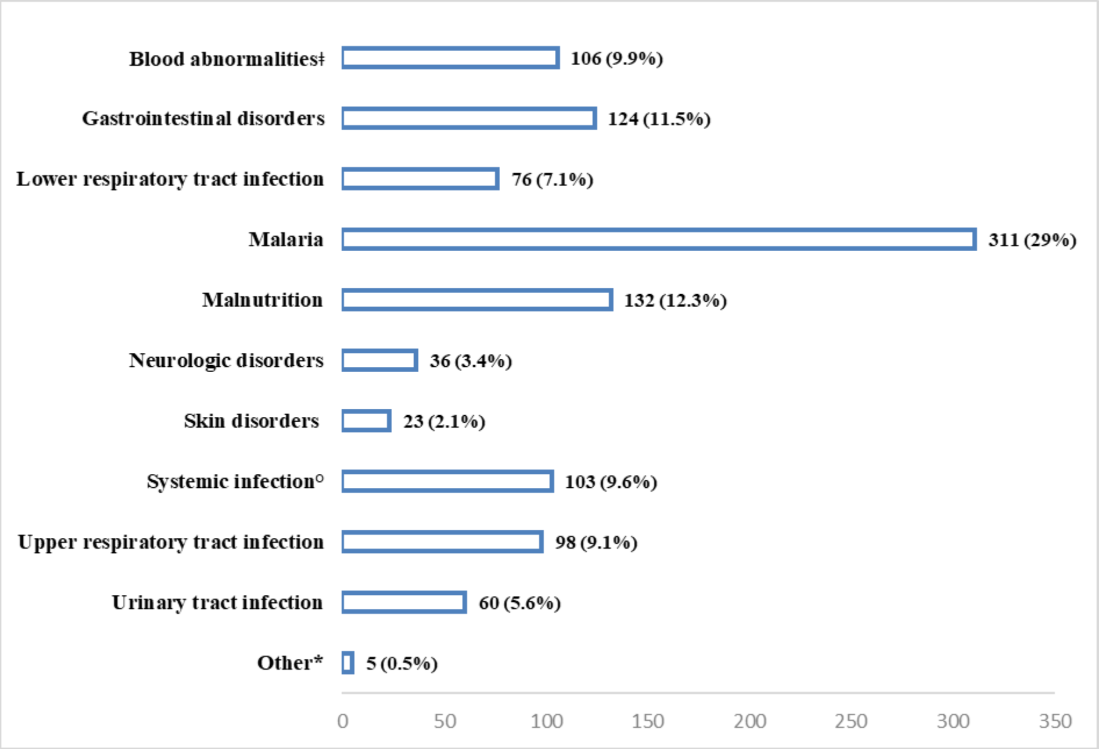

Supplementary Figure S2. Distribution of all the 1,074 diagnoses (MedDRA's preferred terms) among all patients.
‡: cases of anemia only;
*****: injury (n=2), poisoning (n=1), sexual abuse (n=1) and bone infection (n=1);
°: given the malaria burden, systemic infections are presented beside malaria, only for didactic reasons

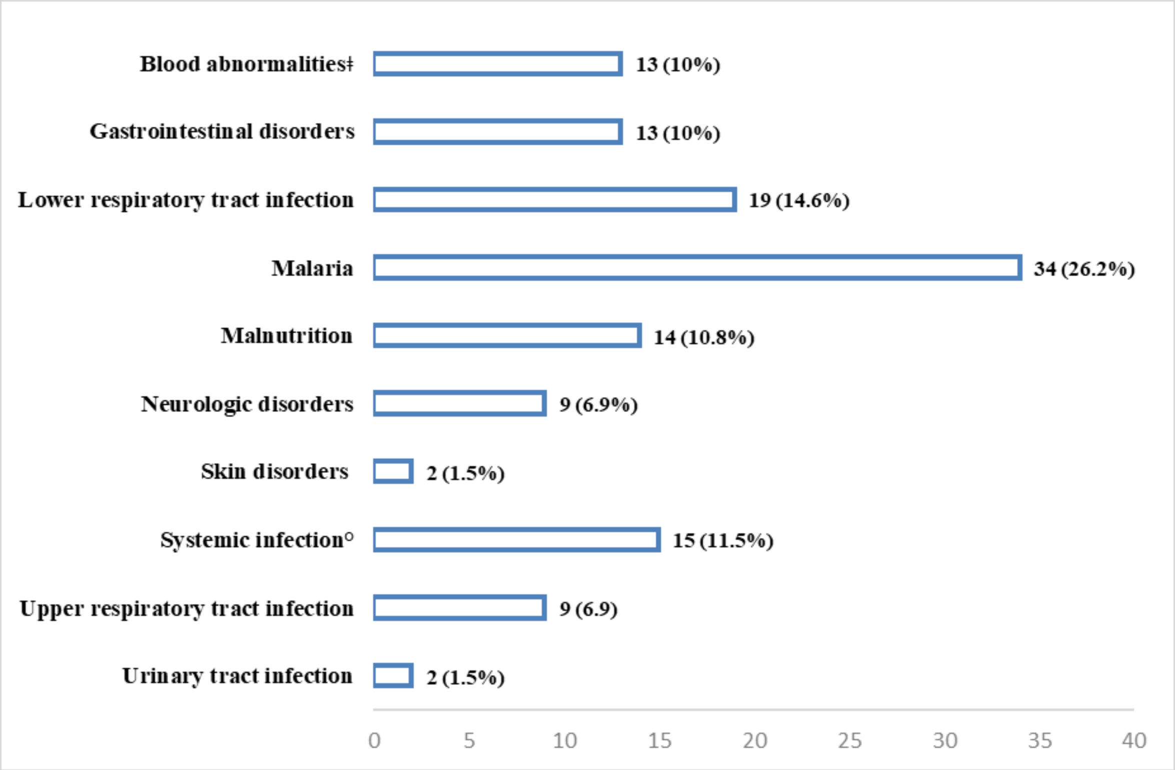

Supplementary Figure S3. Distribution of all the 130 diagnoses (MedDRA's preferred terms) among the patients with danger signs.
‡: cases of anemia only;
°: given the malaria burden, systemic infections are presented beside malaria, only for didactic reasons.

Supplementary Table S1. Diagnostic toolkit overview

Diagnosis	Clinical Presentation Considered For diagnosis	Local results	Diagnostics/ lab results Partners	Specimens	Cooperation partner For analysis
**Upper/Lower Respiratory Tract Infection**					
**Rhinitis**	Respiratory symptoms	Clinical diagnosis	**In children with respiratory signs and meeting criteria for chest x-ray**: viral and bacterial pathogens	Throat swab DNA/RNA	**Influenza A & B**:
**Tracheobronchitis**					**Rhinovirus, Enterovirus, parainfluenza 1-4, coronavirus, hMPV, RSV A & B, Parechovirus, adenovirus:** Institute of Virology, University of Bonn, Germany
**Bronchiolitis**					
**Non-radiological pneumonia**					
**Radiological Pneumonia**		Chest X-ray			
**Severe pneumonia**					***C. pneumoniae, M. pneumoniae, H. influenzae:*** Pharmaceutical Microbiology University Hospital Bonn, University Bonn, Germany
**Gastroenteritis**					
**Salmonellosis**	Diarrhea	Stool culture positive	Confirm Isolates and antibiogram	Isolate	**Confirm all stool Isolates with MALDI and VITEK**: University Hospital Hamburg-Eppendorf (*UKE*) / Institute of Med. Microbiology, University of Münster, Germany
**Campylobacteriosis**		Stool culture positive	Confirm Isolates and antibiogram	Isolate	**FTD PCR for verotoxin *E.coli, Clostridium diff., Yersinia enterocol., Campylobacter, Salmonell*a *spp.***: Hamburg Germany
**Shigellosis**		Stool culture positive	Confirm Isolates and antibiogram	Isolate	
**Rotavirus infection**		Ag-test positive	PCR	NA	**Gastro-Panel PCR for stool viruses** Institute of Virology, University of Bonn, Germany
**Sapovirus infection**			PCR		Bernhard Nocht Institute of Tropical Medicine (BNITM) Hamburg Germany
**Norovirus infection**		Ag-test positive		NA	
**Astrovirus infection**					
**Adenovirus infection**		Ag-test positive		NA	
**Amoebic enteritis**				Stool DNA	
**Giardiasis**					
**Cryptosporidiosis**					
**Gastroenteritis of unknown pathogen**			PCRs for ETEC, EIEC, EAEC, EPEC	Stool DNA	
**Urinary Tract Infection**					
**Enterobacteriaceae infections**	All presentations (culture when abnormalities)	Urine dipstick & Urine culture	Confirm Isolates and antibiogram	Isolate	**Confirmation all identified pathogens** University Hospital Hamburg-Eppendorf (*UKE*) / Institute of Medical Microbiology, University of Münster Germany
**Enterococcal infection**					

**Meningitis/Encephalitis**					
**Viral meningitis**	Suspicion for meningitis		PCRs for HSV 1,2, VZV, Enteroviruses	DNA/RNA from CSF	**CSF Samples for WGS of viral pathogens**<br>Hamburg<br>Germany
**Bacterial meningitis**	Suspicion for meningitis	CSF culture positive	Confirm Isolates and antibiogram, Universal sepsis PCR (16S rRNA Sequencing)	Isolate, DNA/RNA from CSF	**Confirmation all identified pathogens 16s RNA sequencing**<br>University Hospital Hamburg-Eppendorf (*UKE*)<br>Germany
**SYSTEMIC INFECTIONS**					
**Bacterial sepsis/ typhoid fever**	All presentations	Blood culture positive	Confirm Isolates and antibiogram	EDTA Blood, Isolate from blood culture	**Confirmation all identified pathogens *Salmonella* Serotyping**<br>University Hospital Hamburg-Eppendorf (*UKE*) / Institute of Medical Microbiology, University of Münster Germany
**Acute hepatitis a**	All presentations	5 fold increase in ALT / AST	Positive IgM for hepatitis A virus, ELISA	Serum	Institute of Virology<br>University of Bonn<br>Germany
**Acute hepatitis b**		5 fold increase in ALT / AST & Positive HBsAg-Test	Positive IgM for hepatitis B virus, ELISA		
**Acute hepatitis e**		5 fold increase in ALT / AST	Positive IgM for hepatitis E virus, ELISA		
**Q-fever**			PCR for *C. burnetii*	EDTA DNA, Serum	BNITM & University of Gießen (Germany)
**Rickettsiosis**			PCR for *R. felis, R. typhi,* Positive IgM for *R. conorii/africae*	EDTA DNA, Serum	**PCR**<br>Bernhard Nocht Institute for Tropical Medicine<br>Hamburg<br>Germany
**Borreliosis**			PCR for Borrelia spp.	EDTA DNA,	
**Bartonellosis**			PCR for Bartonella spp.	EDTA DNA,	
**Leptospirosis**			PCR	EDTA DNA, Serum	
**Brucellosis**			PCR	EDTA DNA, Serum	**PCR**<br>Bernhard Nocht Institute for Tropical Medicine<br>Hamburg<br>Germany
**Malaria**		Thick and thin smear positive	PCR	EDTA DNA	Institute of Tropical Medicine<br>University of Tübingen<br>Germany
**Dengue**		None	Serum DENV1-4	EDTA DNA, Serum	flavivirus and alphavirus Panel PCR<br>Bernhard Nocht Institute for Tropical Medicine<br>Hamburg<br>Germany
**Chikungunya**			PCR		
**EBV**	All presentations (negative to initial screening)		PCR		Institute of Tropical Medicine<br>University of Tübingen<br>Germany
**CMV**					
**HHV6**					

Supplementary Table S2. Assessment of associations between key signs and symptoms of three diseases

Signs	Malaria			
	OR [95%CI]	p-value	adjusted OR [95%CI]	adjusted p-value
Fever grade 1	0.59 [0.42 – 0.83]	0.002	0.59 [0.42 – 0.83]	0.003
Fever grade 3	3.18 [1.9 – 5.53]	<0.001	3.17 [1.9 – 5.51]	<0.001
Restless	0.58 [0.4 –0.84]	0.004	0.58 [0.4 –0.83]	0.004
Lethargic	2.99 [1.86 – 4.88]	<0.001	2.98 [1.86 - 4.87]	<0.001
Severely wasted	1.85 [1.05 – 3.39]	0.038	1.93 [1.08 – 3.55]	0.030
Unconscious	2.05 [1.18 – 3.74]	0.014	1.04 [0.43 – 2.62]	0.012
Dyspnea	0.54 [0.32 – 0.89]	0.017	0.61[0.23 – 1.59]	0.017
Rhinorrhea	0.46 [0.31 – 0.67]	<0.001	0.44 [0.30 – 0.65]	0.001
Cough	0.34 [0.24 – 0.48]	<0.001	0.34 [0.24 – 0.48]	<0.001
Vomiting	1.96 [1.39 – 2.76]	<0.001	1.18 [0.76 – 1.83]	<0.001
Diarrhoea	0.38 [0.26 – 0.55]	<0.001	0.38 [0.26 – 0.55]	<0.001
Splenomegaly	6.02 [4.08 – 9.04]	<0.001	6.13 [4.13 – 9.23]	<0.001
Hepatomegaly	2.75 [1.87 – 4.09]	<0.001	2.75 [1.87 – 4.10]	<0.001
Convulsions	2.22 [1.32 – 3.85]	0.004	2.28 [1.35 – 3.99]	0.003
	Haemophilus influenzae infection			
	OR [95%CI]	p-value	adjusted OR [95%CI]	adjusted p-value
Diarrhoea	2.93 [1.34 – 6.56]	0.007	3.31[1.48 – 7.64]	0.004
Lethargic	0.42 [0.17 – 1.05]	0.054	0.38[0.15 – 0.98]	0.04
Hepatomegaly	0.33 [0.09 – 0.91]	0.052	0.31[0.09 – 0.89]	0.045
	Epstein Barr virus infection			
	OR [95%CI]	p-value	adjusted OR [95%CI]	adjusted p-value
Cough	3.97 [1.32 –13.25]	0.018	1.03[0.29 – 2.84]	0.015

Supplementary Table S3. Evolution of clinical and biological parameters between study patients in relation to malaria, *H. influenzae*, septicemia, and EBV infections

Pathogens features		Status	Hemoglobin (g/dl)		Hematocrit (%)		Leukocytes ($x10^9/l$)		Thrombocytes ($x10^9/l$)		ALT/AST (IU/l)		Others*	
			**Value**	**p-value**	**Value**	**p-value**	**Value**	**p-value**	**Value**	**p-value**	**Value**	**p-value**	**Value**	**p-value**
**Infections**	***P. falciparum*** **Malaria**	Absent	9.7	<0.001	28.6	<0.001	14.6	<0.001	361	<0.001	31.2	0.3	1.6[ƒ]	<0.001
		Present	8.7		25.2		10.1		147		35.7		0.5[ƒ]	
	Haemophilus influenzae	Absent	9	0.1	25.9	0.002	13.9	0.3	268	0.016	41.8[¥]	<0.001	-	-
		Present	9.8		29.6		12		362		18.9[¥]		-	
	**Bacteremia**	Absent	9	0.07	26.4	0.1	12.2	0.1	235	0.2	59.8[ǂ]	0.045	-	-
		Present	10.3		30		16.3		296		47.4[ǂ]		-	
	**Epstein Barr Virus (EBV)**	Absent	9.7	0.8	28.7	0.4	15.6	0.2	367	0.8	28.7[¥]	0.02	-	-
		Present	9.6		27.4		18.3		357		15.9[¥]		-	

(*) when applicable, the item «Others» could be one of the following variables;

Ŧ: Body temperature (°C);

£: Oxygen saturation [%];

ł: Respiratory rate (pm: per minute);

§: Age (Mo.: Months);

***ƒ*:** Number of stools;

(¥) ALT: Alanine transaminase;

(ǂ) AST: Aspartate transaminase

Supplementary Table S4. STROBE Statement—checklist of items that should be included in reports of cross-sectional study

	Item No	Recommendation
**Title and abstract**	1	(*a*) Indicate the study's design with a commonly used term in the title or the abstract<br>The title describes the study design as **"a cross-sectional hospital-based study"**.
		(*b*) Provide in the abstract an informative and balanced summary of what was done and what was found<br>The abstract describes what was done and found in its **Methods** subsection.
**Introduction**		
Background/rationale	2	Explain the scientific background and rationale for the investigation being reported<br>The background and rationale are described in paragraph 1 of the **Introduction** section.
Objectives	3	State specific objectives, including any prespecified hypotheses<br>The specific objectives of the study are stated in paragraph 2 of the **Introduction** section.
**Methods**		
Study design	4	Present key elements of study design early in the paper<br>The study design is presented in the **Abstract** ["Methods" section] and in the "Study design and setting" subsection of the **Methods** section.
Setting	5	Describe the setting, locations, and relevant dates, including periods of recruitment, exposure, follow-up, and data collection<br>The study setting is described in the "Study design and setting" subsection of the **Methods** section.
Participants	6	(*a*) *Cohort study* - Give the eligibility criteria, and the sources and methods of selection of participants. Describe methods of follow-up<br>Not applicable.<br>*Case-control study* - Give the eligibility criteria, and the sources and methods of case ascertainment and control selection. Give the rationale for the choice of cases and controls<br>Not applicable.<br>*Cross-sectional study* - Give the eligibility criteria, and the sources and methods of selection of participants<br>The selection of participants is described in the "Participants" subsection of the **Methods** section.
		(*b*) *Cohort study* - For matched studies, give matching criteria and number of exposed and unexposed<br>Not applicable.<br>*Case-control study* - For matched studies, give matching criteria and the number of controls per case<br>Not applicable.
Variables	7	Clearly define all outcomes, exposures, predictors, potential confounders, and effect modifiers. Give diagnostic criteria, if applicable<br>The "Variables" subsection of the **Methods** section refers to the **Supplementary Table S1** that defines all variables and a full diagnostic toolkit of the study.
Data sources/ measurement	8*	For each variable of interest, give sources of data and details of methods of assessment (measurement). Describe comparability of assessment methods if there is more than one group<br>The "Variables" subsection of the **Methods** section refers to the **Supplementary Table S1** that defines all variables and a full diagnostic toolkit of the study.
Bias	9	Describe any efforts to address potential sources of bias<br>Our efforts in order to minimize both selections and confounding biases were explained in paragraph 3 of the "Data sources and bias assessment" subsection and in paragraph 3 of the "Data management and statistical methods" subsection of the **Methods** section, respectively.
Study size	10	Explain how the study size was arrived at<br>The study size was explained in the "Sample size considerations" subsection of the **Methods** section.
Quantitative variables	11	Explain how quantitative variables were handled in the analyses. If applicable, describe which groupings were chosen and why<br>The way quantitative variables were handled is discussed in paragraph 3 of the "Data management and statistical methods" subsection of the **Methods** section.
Statistical methods	12	(*a*) Describe all statistical methods, including those used to control for confounding<br>Statistical methods are described in the "Data management and statistical methods" subsection of the **Methods** section.
		(*b*) Describe any methods used to examine subgroups and interactions<br>Analytical methods are described in paragraph 3 of the "Data management and statistical methods" subsection of the **Methods** section.
		(*c*) Explain how missing data were addressed<br>The way missing data were handled is described in paragraph 1 of the "Data management and statistical methods" subsection of the **Methods** section.
		(*d*) *Cohort study* - If applicable, explain how loss to follow-up was addressed<br>Not applicable.<br>*Case-control study* - If applicable, explain how matching of cases and controls was addressed<br>Not applicable.

		Cross-sectional study - If applicable, describe analytical methods taking account of sampling strategy<br>Analytical methods are described in paragraph 3 of the "Data management and statistical methods" subsection of the **Methods** section.
		(*e*) Describe any sensitivity analyses<br>Not applicable.
**Results**		
Participants	13*	(a) Report numbers of individuals at each stage of study - e.g. numbers potentially eligible, examined for eligibility, confirmed eligible, included in the study, completing follow-up, and analysed<br>The number of participants at each level completing each phase of data collection is shown in **Fig. 1**.
		(b) Give reasons for non-participation at each stage<br>Not applicable.
		(c) Consider use of a flow diagram<br>**Figure 1**. Flow diagram of signs and symptoms, laboratory findings, and diagnoses in all patients throughout the study.
Descriptive data	14*	(a) Give characteristics of study participants (e.g. demographic, clinical, social) and information on exposures and potential confounders<br>Characteristics of the study participants are presented in the "Study patients" subsection of the **Results** section and summarized in **Table 1**.
		(b) Indicate number of participants with missing data for each variable of interest<br>The number of participants with missing data is indicated in parentheses throughout the **Results** section.
		(c) *Cohort study* - Summarise follow-up time (eg, average and total amount)<br>Not applicable.
Outcome data	*15**	*Cohort study* - Report numbers of outcome events or summary measures over time<br>Not applicable.
		Case-control study - Report numbers in each exposure category, or summary measures of exposure<br>Not applicable.
		Cross-sectional study - Report numbers of outcome events or summary measures<br>Numbers (along with percentages) of outcome events are reported throughout the **Results** section.
Main results	16	(*a*) Give unadjusted estimates and, if applicable, confounder-adjusted estimates and their precision (e.g. 95% confidence interval). Make clear which confounders were adjusted for and why they were included<br>Unadjusted estimates and their precision are given, and the confounders for which adjustments were made for are presented in paragraph 1 of the "More frequent infections and their characteristics" subsection of the **Results** section.
		(*b*) Report category boundaries when continuous variables were categorized<br>Not applicable.
		(*c*) If relevant, consider translating estimates of relative risk into absolute risk for a meaningful time period<br>Not applicable.
Other analyses	17	Report other analyses done – e.g. analyses of subgroups and interactions, and sensitivity analyses<br>Both analyses of subgroups and interactions are reported in the **Results** section, **Table 2**, and **Supplementary Tables S2 &S3**.
**Discussion**		
Key results	18	Summarise key results with reference to study objectives<br>Results are summarized in paragraphs 1-13 of the **Discussion** section.
Limitations	19	Discuss limitations of the study, taking into account sources of potential bias or imprecision. Discuss both direction and magnitude of any potential bias<br>Limitations are discussed in paragraph 14 of the **Discussion** section.
Interpretation	20	Give a cautious overall interpretation of results considering objectives, limitations, multiplicity of analyses, results from similar studies, and other relevant evidence<br>Paragraphs 14 & 15 of the **Discussion** section.
Generalisability	21	Discuss the generalisability (external validity) of the study results<br>Paragraph 15 and **Conclusion** section.
**Other information**		
Funding	22	Give the source of funding and the role of the funders for the present study and, if applicable, for the original study on which the present article is based<br>Sources of funding and the role of funders of the current study are provided in the submission form.

Supplementary Table S5. Detailed study case definitions

Items	Definitions/descriptions
**Fever**	• Rectal temperature ≥38°C OR<br>• the axillary temperature at one of two measured sides ≥38°C → the higher temperature measured will be taken into account
**Diarrhea**	• A state in which an individual experiences a change in normal bowel habits characterized by the frequent passage of loose, liquid, unformed stools (three or more loose stools within a 24-hour period) AND<br>• The collected stool sample is loose/watery (inspection)
**Dyspnea**	ONE of the following signs<br>• Intercostal retractions/chest indrawing<br>• Stridor in calm child<br>• Tachypnea<br>○ Younger than two months: >60 breaths/min<br>○ Two to 12 months: >50 breaths/min<br>○ One to 5 years: >40 breaths/min<br>○ ≥5 years: >20 breaths/min
**Respiratory distress**	ONE of the following signs<br>• Difficult breathing<br>• Hypoxemia (pulse oximetry <95%)<br>• Physical examination with signs of respiratory distress (*i.e.* Definite crackles or wheezing in auscultation)
**Signs of upper respiratory tract infection (URTI)**	ONE of the following signs<br>• Cough<br>• Nasal discharge/rhinorrhea<br>• Nasal congestion<br>• Sneezing<br>• Sore throat
**Signs/symptoms of lower respiratory tract infection (LRTI)**	ONE of the following signs<br>• Cough<br>• Chest pain<br>• Respiratory distress (see above)
**Respiratory signs/symptoms**	• Respiratory distress<br>• Signs of upper respiratory tract infection (URTI)<br>• Signs of lower respiratory tract infection (LRTI)
**Urinary tract signs/symptoms**	ONE of the following signs<br>• Dysuria during the last 24h (reported)<br>• Urine abnormal during the last 24h (reported)<br>• Urine strips positive (Nitrite OR Leukocytes increased)<br>• Physical examination with signs of urinary tract infection (*i.e*. renal angle tenderness)
**Neurological signs/symptoms**	ONE of the following signs<br>• Convulsions/Seizures during the last 24h (reported)<br>• Stiff neck<br>• Kernig's sign (children > 1 y)<br>• Bulging fontanelles (children < 3 months)<br>• Physical examination with signs of neurological infection
**Abdominal signs**	ONE of the following signs<br>• Jaundice (except neonatal icterus)<br>• Ascites<br>• Hepatomegaly<br>• ALT or AST pathologically increased (>50 U/L, infants <12 months: >80 U/L)<br>• Physical examination with signs of liver inflammation
**Danger signs***	ONE of the following signs:<br>• Coma (BCS≤2)<br>• Chest indrawing<br>• Prostration

Supplementary Table S6. Variables captured by the study questionnaire*

**Exposure and General Condition**		
Previous Drug Intake<br>- Antimalarials?<br>- Antibiotics?<br>- Antipyretics?<br>If yes, what?<br>Medical History<br>- Was the child admitted to any hospital in the last 6 months?<br>- Does the child have any underlying disease?<br>Immunization Check<br>- If child's health card is available, a picture of vaccinations is taken	Is/was the child (last 24h) …<br>- Restless/irritable?<br>- Weak/lethargic?<br>- Unable to drink?<br>- Unable to eat?<br>- Wasted?<br>- Prostrated?<br>- Unconscious?	Blantyre coma score<br>Does/did the child have (last 24h) …<br>- A rash?<br>- A wound infection?<br>- A skin/soft tissue infection?<br>If yes, which?<br>- A generalized/localized lymphadenopathy<br>If yes, where?<br>***Otoscopy examination:***<br>- Normal findings/otitis<br>- Swelling behind the ear?<br>***Throat inspection:***<br>- Normal findings/red pharynx /purulent tonsils?<br>***Cardiac examination:***<br>- Heart rate<br>- Heart murmur?
**Respiratory Condition**		
Does/did the child have (last 24h) …<br>- Difficult breathing?<br>- Frequent sneezing?<br>- A sore throat<br>- A blocked nose<br>- Nasal discharge/rhinorrhea?<br>- A cough?<br>Is sputum productive?	Does the child have…<br>- Intercostal retractions?<br>- Chest indrawing?<br>- Nose flaring?<br>- A stridor?	***Auscultation:***<br>Is/Are There…<br>- Diminished/missing breath sounds?<br>- Crackles?<br>- Bronchial breath sounds?<br>- Wheezing?<br>Breath rate<br>Oxygen saturation
**Gastrointestinal Condition**		
Does/did the child have (last 24h) …<br>- Vomiting?<br>- Vomiting everything it takes?<br>- Loose stool?<br>If yes, how often?<br>- Bloody/Mucoid stool?	***Auscultation:***<br>Bowel sounds…<br>- Missing?<br>- Hyper-peristaltic?	***Inspection:***<br>- Jaundice (> 1 Month)?<br>- Ascites<br>***Palpation:***<br>- Muscular guarding?<br>- Abdominal mass?<br>- Spleen enlargement?<br>- Liver enlargement?<br>- Abdominal pain?<br>If yes, McBurney positive?
**Urinary Condition**		
Does/did the child have (last 24h) …<br>- Dysuria?<br>- Abdominal urine?	***Physical examination:***<br>Does the child have…<br>- Renal angle tenderness?	
**Neurological Condition**		
Does/did the child have (last 24h…)<br>- Convulsions/Seizures?	***Physical Examination:***<br>Does the child have…<br>- A stiff neck?<br>- A positive Kernig's sign (> 1 yr)?<br>- Bulging fontanelle (<3 Months)?	

(*): The different items (including the questions) of the study questionnaire are categorized under subtitles such as exposure, general/respiratory /gastrointestinal /urinary/neurological conditions.

Supplementary Table S7. Specification of laboratory examinations performed in Lambaréné and by partners

Samples	Lambaréné			Partners		
	Volume	Laboratory Test	Diagnosis	Volume	Laboratory Test	Diagnosis
**EDTA Blood**	25 µl	Malaria parasite count	Malaria	100 µl (DNA/RNA)	qPCR	Bacteria:<br>*S. agalactiae*; *L. monocytogenes; E. coli; S. aureus; C. trachomatis S. pneumonia, C. burnetii; Salmonella spp.; Brucella* spp.; *U. urealyticumparvum; Leptospira* spp.*;* *Rickettsia* spp.<br>Viruses:<br>Human herpesvirus 6; Epstein Barr virus; Cytomegalovirus; Dengue virus 1-4; Chikungunya virus; West Nile virus; Yellow fever virus Parasites:<br>*Plasmodium* spp., *Babesia* spp.
	1 ml	Full Blood Count	Anemia, Leukocytosis	NA	NA	
**Native blood**	1 – 3 ml	Blood culture	Systemic bacterial Infection/sepsis	NA	NA	NA
**Full blood or serum**	100 µl	HIV RDT test	HIV	NA	NA	NA
**Serum**	100 µl	ALT & AST	Liver function	NA	NA	NA
	75 µl	AgHBs	Hepatitis B virus	200 µl (DNA/RNA)	qPCR	NA
	500 µl	Antibodies	Viruses;<br>Bacteria<br>(not detectable by culture)			
**Saliva**	NA	NA	NA	100 µl (DNA/RNA)	qPCR	Bacteria:<br>*Chlamydiae Haemophilus influenzae; M. pneumonia*<br>Viruses:<br>Influenza A & B; Rhinovirus; Coronavirus (NL63, 229E, OC43 & HKU1); Human parainfluenza virus type 1, 2, 3 & 4; Human metapneumovirus; Respiratory syncytial virus type A & B; Adenovirus; Enterovirus; Parechovirus
**Urine**	10 µl	Stick	Leukocytes<br>Nitrites	NA	NA	NA

**Urine**	2 ml	Culture	*Enterobacterales, non-fermenting Gram-negative bacteria*<br>*S. aureus, S. saprophyticus*<br>Other bacteria	NA	NA	NA
	100 µl	Legionella Ag test	*L. pneumophila*			
**Stool**	Pea size	RDT stool	Rotavirus; Adenovirus; Norovirus	200 µl (DNA)	qPCR	<u>Viruses:</u><br>Norovirus group 1 & 2; Astrovirus; Rotavirus; Adenoviruses; Sapovirus; Enterovirus; Parechovirus
		Culture	*Salmonella* spp.*;* *Campylobacter* spp.*;* *Shigella* spp*;*<br>Other bacteria			
**CSF**	4 ml	CSF microscopy culture	*S. pneumoniae;*<br>*E. coli*<br>*B. streptococcus*, *N. meningitis B; H. influenzae*	0.5 ml (DNA/RNA)	qPCR	<u>Viruses:</u><br>Herpes Simplex Virus type 1 & 2 ; Varicella-zoster virus; Enterovirus; Mumps virus; Parechovirus<br><u>Bacteria:</u><br>*S. agalactiae*; *L. monocytogenes*; *E. coli*

This table presents for each type of specimen (blood, serum, nasal swabs, urine, stool, and CSF, the optimal volumes/size), the lab tests to be performed for pathogens identification/detection either in Lambaréné and/or partners laboratories.

Supplementary Table S8. Differences in clinical parameters among study patients in relation to major clinical signs with p-values

Clinical features		All participants	Status	Age (Months)		Body temperature (°C)		Number of stools		Respiratory rate (per minute)	
		N (%)		Value	p-value	Value	p-value	Value	p-value	Value	p-value
General signs	Restless	182 (30.6)	Absent	-	-	-	-	-	-	44	< 0.001
			Present	-		-		-		49	
	Wasted	63 (10.6)	Absent	-	-	-	-	1	0.048	-	-
			Present	-		-		0.7		-	
HEENT	Rhinorrhea	159 (27)	Absent	-	-	-	-	-	-	44	< 0.001
			Present	-		-		-		50	
Gastro-intestinal	Vomiting	326 (54.3)	Absent	-	-	-	-	0.6	< 0.001	-	-
			Present	-		-		1.3		-	
	Diarrhea	188 (32)	Absent	-	-	-	-	0	< 0.001	-	-
			Present	-		-		3		-	
	Bloody stool	69 (12)	Absent	-	-	-	-	0.6	< 0.001	-	-
			Present	-		-		3		-	
	Abdominal pain	109 (18.4)	Absent	-	-	-	-	-	-	47	< 0.001
			Present	-		-		-		41	
Neurological	Prostrated	23 (4)	Absent	-	-	39	< 0.001	-	-	-	-
			Present	-		38.6		-		-	
	Unconscious	66 (11)	Absent	47	0.041		-	-	-	-	-
			Present	36				-		-	
Respiratory	Frequent sneezing	6 (1)	Absent	-	-	39	0.02	-	-	-	-
			Present	-		38.3		-		-	
	Cough	270 (46)	Absent	-	-	-	-	-	-	42	< 0.001
			Present	-		-		-		50	
	Bronchial breath sounds	105 (18.4)	Absent	-	-	-	-	-	-	44	< 0.001
			Present	-		-		-		53	
	Flaring	63 (11)	Absent	-	-	-	-	-	-	44	< 0.001
			Present	-		-		-		58	
	Sore throat	5 (0.8)	Absent	-	-	-	-	-	-	45	0.011
			Present	-		-		-		61	
Urinary	Pain in passing urine	14 (2.4)	Absent	-	-	-	-	-	-	46	0.011
			Present	-		-		-		36	
	Increased frequency of urination	11 (1.9)	Absent	47	< 0.001	-	-	-	-	-	-
			Present	20		-		-		-	

HEENT: head, eyes, ears, nose, and throat;

Supplementary Table S9. Differences in biomedical parameters among study patients in relation to major clinical signs with p-values

Clinical features		All participants	Status	Hemoglobin (g/dL)		Hematocrit (%)		Leukocytes ($x10^9/L$)		Thrombocytes ($x10^9/L$)		Transaminases* (IU/L)	
		N (%)		Value	p-value	Value	p-value	Value	p-value	Value	p-value	Value	p-value
General signs	Restless	182 (30.6)	Absent	9.1	0.9	26.2	0.5	11.9	0.1	216	<0.001	33.7	0.9
			Present	9.1		26.7		13.2		280		33.4	
	Lethargic	499 (83.9)	Absent	9.9	0.002	28.9	0.002	12.6	0.7	297	<0.001	49.2‡	0.004
			Present	8.9		25.9		12.2		224		61.4‡	
	Wasted	63 (10.6)	Absent	9.2	0.02	26.6	0.031	12.2	0.5	239	0.4	33	0.5
			Present	8.3		24		13.0		212		37.7	
HEENT	Rhinorrhea	159 (27)	Absent	9.2	0.06	26.2	0.3	11.8	0.1	221	0.003	34.8	0.5
			Present	8.4		27		13		275		30.9	
Gastro-intestinal	Vomiting	326 (54.3)	Absent	8.8	0.02	25.6	0.02	12.9	0.1	252	0.07	34.4	0.7
			Present	9.3		27.1		11.7		223		32.6	
	Diarrhea	188 (32)	Absent	8.8	<0.001	25.6	0.002	12.3	0.8	210	<0.001	33.9	0.5
			Present	9.6		27.9		12.1		292		31.3	
	Bloody stool	69 (12)	Absent	-	-	-	-	-	-	-	-	-	-
			Present										
	Abdominal pain	109 (18.4)	Absent	9.1	0.9	26.4	0.8	12.3	0.7	239	0.3	33.4	0.8
			Present	9.1		26.1		11.9		218		32.6	
	Hepatomegaly	199 (35)	Absent	9.5	<0.001	27.8	<0.001	11.6	0.1	268	<0.001	55.3‡	0.024
			Present	8.1		23.5		13.4		175		67.6‡	
Neurological	Convulsion	80 (13.5)	Absent	9.1	0.1	26.6	0.06	13.3	0.624	244	0.007	32.1	0.169
			Present	8.6		24.6		11.9		186		40.6	
	Prostrated	23 (4)	Absent	9.1	0.4	26.4	0.4	12.2	0.5	237	0.53	33.6	
			Present	8.6		24.8		13.5		211		32.6	
	Unconscious	66 (11)	Absent	9.1	0.06	26.6	0.1	12.2	0.1	244	0.008	32.9¥	0.4
			Present	8.5		24.4		12.8		175		38.9¥	
Respiratory	Frequent sneezing	6 (1)	Absent	9.1	0.1	26.4	0.007	12.2	1	234	0.004	33.8¥	0.013
			Present	10.9		35		12.4		447		22.3¥	
	Cough	270 (46)	Absent	9	0.3	26.1	0.3	11.4	0.031	201	<0.001	54.9	0.032
			Present	9.2		26.8		13.08		278		65.4	
	Crackles	66 (11.5)	Absent	9	0.2	26.2	0.1	11.7	0.044	227	0.001	32.7	0.4
			Present	9.6		27.9		14.7		306		37.7	
	Bronchial breath sounds	105 (18.4)	Absent	9	0.4	26.4	0.7	11.4	0.005	221	<0.001	33.8	0.6
			Present	7.6		26.7		14.3		300		16	
	Flaring	63 (11)	Absent	9.1	0.4	26.7	0.033	11.7	0.043	234	0.6	32	0.1
			Present	8.8		24.4		15.2		246		51.2	
	Sore throat	5 (0.8)	Absent	9	<0.001	26.4	0.002	12.1	0.3	234	0.004	33.8	0.6
			Present	10.7		34.2		15.4		447		23	

Urinary	Pain in passing urine	14 (2.4)	Absent	9.1	0.6	26.4	0.8	12.2	0.01	239	<0.001	-	-
			Present	9.4		25.7		8.3		137		-	
	Increased frequency of urination	11 (1.9)	Absent	9.1	0.6	26.5	0.4	12.1	0.5	241	0.001	32.9	0.025
			Present	8.7		24.5		10.3		112		21	
Lymphatic	Splenomegaly	253 (44.2)	Absent	9.9	<0.001	28.8	<0.001	12.1	0.7	296	<0.001	53.6ǂ	0.006
			Present	7.9		23.1		12.4		161		66.9ǂ	

(*) Transaminases: are reflecting ALT values except when specified by a "**ǂ"** it is rather AST: Aspartate transaminase;

HEENT: head, eyes, ears, nose, and throat.
